# Gut Microbiota Contribution to Weight-Independent Glycemic Improvements after Gastric Bypass Surgery

**DOI:** 10.1128/spectrum.05109-22

**Published:** 2023-04-06

**Authors:** Mohammed K. Hankir, Petia Kovatcheva-Datchary, Rebecca Springer, Annett Hoffmann, Jörg Vogel, Florian Seyfried, Tulika Arora

**Affiliations:** a Department of General, Visceral, Transplantation, Vascular and Pediatric Surgery, University Hospital Wurzburg, Wurzburg, Germany; b Institute for Molecular Infection Biology, University of Wurzburg, Wurzburg, Germany; c Department of Pediatrics, University Hospital Wurzburg, Wurzburg, Germany; d Helmholtz Institute for RNA-based Infection Research, Helmholtz Centre for Infection Research, Wurzburg, Germany; e Novo Nordisk Foundation Center for Basic Metabolic Research, Faculty of Health and Medical Sciences, University of Copenhagen, Copenhagen, Denmark; Lerner Research Institute

**Keywords:** gastric bypass surgery, glycemic control, gut microbiota, caloric restriction, germfree mice

## Abstract

Roux-en-Y gastric bypass surgery (RYGB) leads to improved glycemic control in individuals with severe obesity beyond the effects of weight loss alone. Here, We addressed the potential contribution of gut microbiota in mediating this favourable surgical outcome by using an established preclinical model of RYGB. 16S rRNA sequencing revealed that RYGB-treated Zucker fatty rats had altered fecal composition of various bacteria at the phylum and species levels, including lower fecal abundance of an unidentified *Erysipelotrichaceae* species, compared with both sham-operated (Sham) and body weight-matched to RYGB-treated (BWM) rats. Correlation analysis further revealed that fecal abundance of this unidentified *Erysipelotrichaceae* species linked with multiple indices of glycemic control uniquely in RYGB-treated rats. Sequence alignment of this *Erysipelotrichaceae* species identified Longibaculum muris to be the most closely related species, and its fecal abundance positively correlated with oral glucose intolerance in RYGB-treated rats. In fecal microbiota transplant experiments, the improved oral glucose tolerance of RYGB-treated compared with BWM rats could partially be transferred to recipient germfree mice, independently of body weight. Unexpectedly, providing *L. muris* as a supplement to RYGB recipient mice further improved oral glucose tolerance, while administering *L. muris* alone to chow-fed or Western style diet-challenged conventionally raised mice had minimal metabolic impact. Taken together, our findings provide evidence that the gut microbiota contributes to weight loss-independent improvements in glycemic control after RYGB and demonstrate how correlation of a specific gut microbiota species with a host metabolic trait does not imply causation.

**IMPORTANCE** Metabolic surgery remains the most effective treatment modality for severe obesity and its comorbidities, including type 2 diabetes. Roux-en-Y gastric bypass (RYGB) is a commonly performed type of metabolic surgery that reconfigures gastrointestinal anatomy and profoundly remodels the gut microbiota. While it is clear that RYGB is superior to dieting when it comes to improving glycemic control, the extent to which the gut microbiota contributes to this effect remains untested. In the present study, we uniquely linked fecal *Erysipelotrichaceae* species, including Longibaculum muris, with indices of glycemic control after RYGB in genetically obese and glucose-intolerant rats. We further show that the weight loss-independent improvements in glycemic control in RYGB-treated rats can be transmitted via their gut microbiota to germfree mice. Our findings provide rare causal evidence that the gut microbiota contributes to the health benefits of metabolic surgery and have implications for the development of gut microbiota-based treatments for type 2 diabetes.

## OBSERVATION

The global prevalence of obesity and its associated comorbidities such as type 2 diabetes and fatty liver disease continues to rise (1). While the pathogenesis of obesity is complex and multifactorial (2), evidence suggests that obesity-associated gut microbiotas causally promote weight gain through increased energy harvest from food (3). Of the treatments that are currently available for severe obesity, metabolic surgeries such as Roux-en-Y gastric bypass (RYGB) and vertical sleeve gastrectomy (VSG) remain by far the most effective (4). These surgical procedures were originally thought to exert their beneficial metabolic effects purely through mechanical processes such as physical restriction of food intake due to a smaller stomach (as is the case for both RYGB and VSG) and/or malabsorption of ingested food due to redirection of nutrient flow to the distal small intestine (as is the case for RYGB) (5). It is now clear, however, that distinct molecular, cellular, and system-wide changes occur after metabolic surgery (6). This includes pronounced shifts in gut microbiota—particularly for RYGB—such as decreased *Firmicutes* and increased *Proteobacteria* abundances at the phylum level (7). Accumulating evidence further suggests that the gut microbiota plays a causal role in some of the health benefits associated with metabolic surgery. For example, the weight loss and improvements in glycemic control normally found in RYGB-treated and VSG-treated rats or mice, respectively, with diet-induced obesity is attenuated by prior depletion of the gut microbiota with antibiotic treatments (8, 9). Additionally, transfer of the cecal/fecal microbiota from metabolic surgery-treated mice (10), rats (11, 12) or humans (13, 14) to germfree (GF) mice can lead to weight loss and protection from fat gain (10, 13) or to improvements in glycemic control in recipients (11, 12, 14). These studies, however, do not disentangle the potential influences that the gut microbiota, body weight, and glycemia have on each other. Specifically, the weight gain from antibiotic treatment in RYGB-treated rats/VSG-treated mice could itself worsen glycemic control. On the other hand, the weight loss after metabolic surgery could cause shifts in the gut microbiota that in turn promote improvements in glycemic control. To circumvent these issues, we reanalyzed the fecal microbiotas of Zucker fatty rats that underwent sham operations (Sham), that were RYGB treated, and that were body weight matched to RYGB-treated rats (BWM) in relation to indices of glycemic control during an oral glucose tolerance test (OGTT) (15, 16). We then transplanted fecal samples from RYGB-treated and BWM rats to GF mice and evaluated the impact on body weight and oral glucose tolerance in recipients.

The metabolic phenotype of the Zucker fatty rats used in the present study has partially been reported in previous studies (15, 16). RYGB-treated and BWM rats had similar reductions in body weight, in contrast to Sham rats by the end of the 28-day postoperative observation period (see Table S1 in the supplemental material). Notably, BWM rats had to be given less food than RYGB-treated rats to achieve a similar body weight, which has been attributed to increased energy expenditure after metabolic surgery (17) (Table S1). Importantly, RYGB-treated rats had improved glycemic control compared to both Sham and BWM rats, as reflected by lower areas under the curve (AUC) from OGTT, a higher Matsuda-DeFronzo insulin sensitivity index (ISI-M), and a lower homeostatic model of insulin resistance (HOMA-IR) at postoperative day 27 (Table S1).

**FIG 1:**
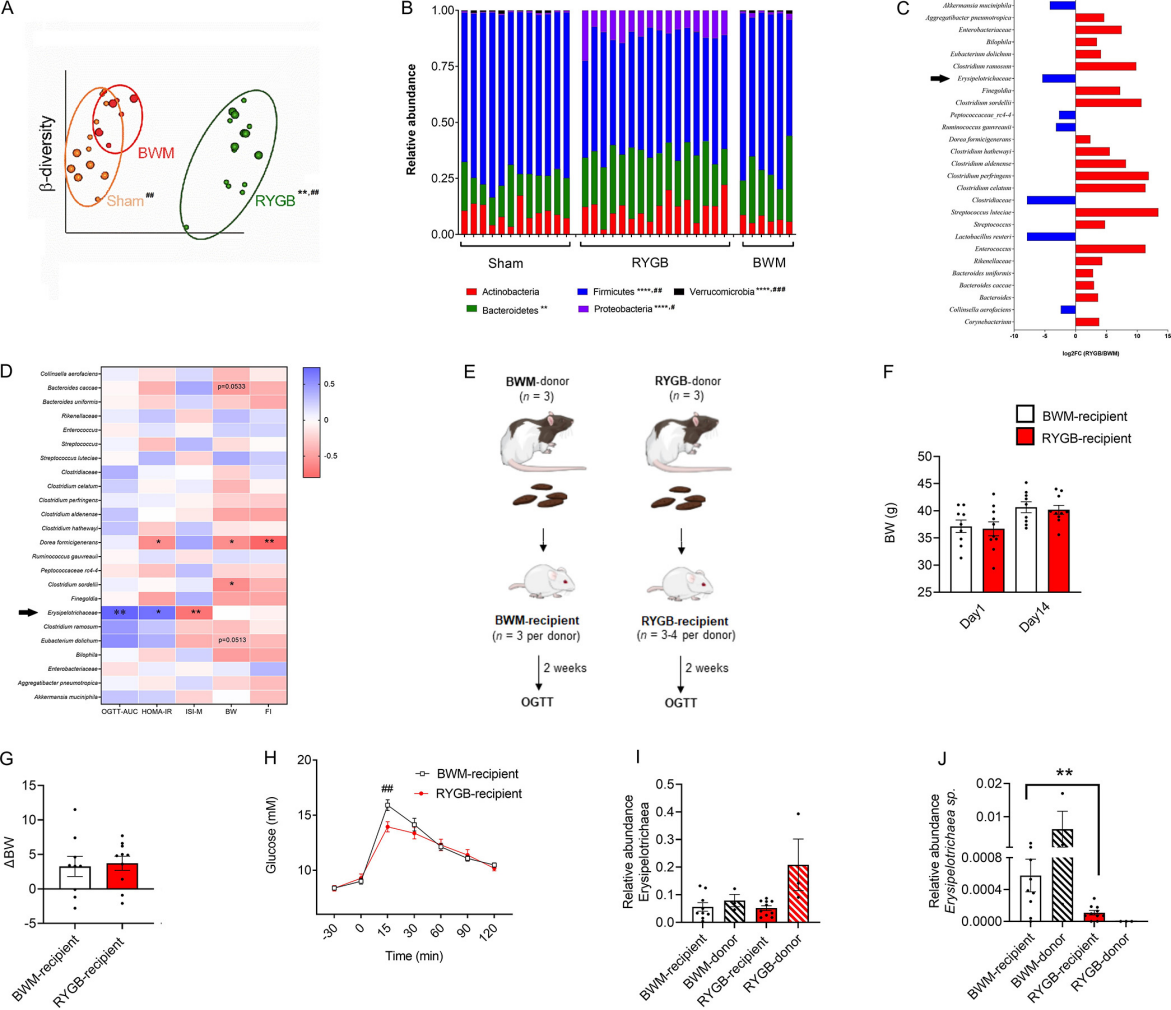
Evidence that the gut microbiota contributes to the weight loss-independent improvement in glycemic control after RYGB. (A) Beta-diversity plot, measured by Bray-Curtis analysis, showing separation of the fecal microbiota in Sham (*n *= 12) and BWM (*n *= 5) rats from that in RYGB-treated (*n *= 16) rats at postoperative day 28. **, *Q* < 0.01 versus Sham rats, and ##, *Q* < 0.01, versus BWM rats, as determined by PERMANOVA followed by a pairwise *post hoc* test. (B) Relative abundance of phyla in Sham (*n *= 12), RYGB-treated (*n *= 16), and BWM (*n *= 5) rats. ****, *Q* < 0.0001, and **, *Q* < 0.01, for RYGB-treated versus Sham rats, and ###, *P* < 0.001, ##, *P* < 0.01, and #, *P* < 0.05, for RYGB-treated versus BWM rats, as determined by the Kruskal-Wallis test corrected for FDR by the Benjamini-Hochberg method. (C) Differential species abundance, expressed as log_2_ fold change in RYGB-treated versus BWM rats. (D) Correlation matrix of fecal microbiota species with metabolic parameters in RYGB-treated rats. **, *Q* < 0.01, and *, *Q* < 0.05, as determined by the Mann-Whitney U test after adjusting for multiple comparisons. (E) Schematic diagram showing protocol for fecal microbiota transplant in GF mice. (F to H) Body weights at day 1 and day 14 (F), body weight difference (G), and OGTT (H) in GF mice (*n *= 9 or 10/group) transplanted with feces from BWM (*n *= 3) and RYGB-treated (*n *= 3) rats. ##, *P* < 0.01, as determined by two-way ANOVA followed by Bonferroni’s *post hoc* test. (I and J) Relative fecal abundance of the family *Erysipelotrichaceae* (I) and an unidentified species belonging to the family *Erysipelotrichaceae* (J) in recipient GF mice (*n *= 9 or 10/group) transplanted with feces from BWM (*n *= 3) and RYGB-treated (*n *= 3) rats. ##, *P* < 0.01, as determined by the Mann-Whitney U test.

Fecal samples were collected from all groups on postoperative day 28 and were later subjected to 16S rRNA sequencing. Principal-coordinate analysis (PCoA) revealed that Sham and BWM rats had comparable beta-diversity, while RYGB-treated rats clustered separately (Fig. 1A). At the phylum level, RYGB-treated rats had higher fecal abundance of *Proteobacteria*, while *Firmicutes* and *Verrucomicrobia* were lower than in Sham and BWM rats (Fig. 1B). At the species level, fecal abundances of 31 bacteria were lower in RYGB-treated rats compared with Sham rats, while 11 were higher (Fig. S1A). On the other hand, fecal abundances of 20 bacterial species were higher in RYGB-treated rats than BWM rats, while abundances of 7, including Akkermansia muciniphila, Lactobacillus reuteri, and Collinsella aerofaciens, were lower (Fig. 1C). In contrast, fecal abundances of 12 bacterial species were lower in Sham rats than BWM rats, while abundances of 9 were higher (Fig. S1B). An unidentified species belonging to the family *Erysipelotrichaceae*, a Gram-positive bacterium from the phylum *Firmicutes* previously linked to inflammatory bowel and metabolic disease (18), was lower in RYGB-treated rats than both Sham and BWM rats (Fig. 1C). Further, this unidentified species positively correlated with OGTT AUC and HOMA-IR and negatively correlated with ISI-M uniquely in RYGB-treated rats (Fig. 1D). Additional correlation analysis revealed that fecal *Rikenellaceae* species positively correlated with HOMA-IR and negatively correlated with ISI-M, body weight, and food intake in Sham rats (Fig. S1C) and body weight in BWM rats (Fig. S1D).

To determine the potential causal role of the gut microbiota in weight loss-independent improvements in glycemic control after RYGB, fecal microbiota transplant experiments were performed on GF mice followed 2 weeks later by an OGTT (Fig. 1E). Two groups of GF mice with similar starting body weights were subjected to oral gavage with fecal slurries from RYGB-treated and BWM rats (Fig. 1F). While there was no difference in body weight gain between the two recipient groups (Fig. 1G), blood glucose levels during the OGTT were significantly lower in RYGB recipient mice at the 15-min time point (Fig. 1H). Analysis of the composition of fecal microbiotas of donors and recipients revealed no overall difference in the abundance of members of the family *Erysipelotrichaceae* between groups (Fig. 1I). However, the unidentified *Erysipelotrichaceae* species that was depleted in the feces of RYGB donors compared with BWM donors followed a similar pattern in the feces of the corresponding groups of recipient mice (Fig. 1J).

**FIG 2:**
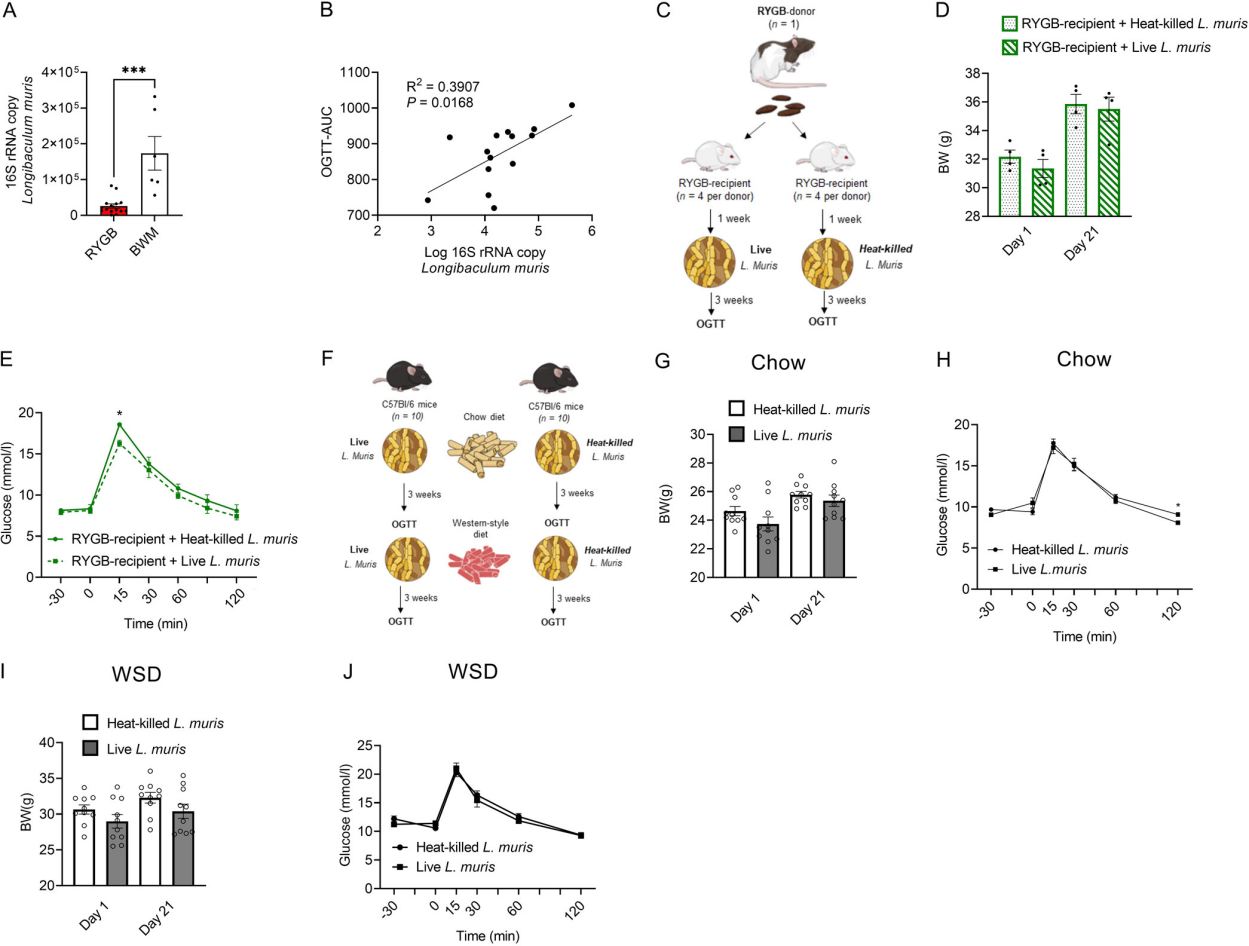
Metabolic impact of *L. muris* on RYGB-recipient and conventionally raised mice. (A) qPCR analysis of Longibaculum muris in feces of BWM (*n *= 5) and RYGB-treated (*n *= 16) rats at postoperative day 28. ***, *P* < 0.001, as determined by the Mann-Whitney U test. (B) Correlation between abundance of fecal *L. muris* and OGTT AUC in RYGB-treated rats. (C) Protocol for *L. muris* administration in RYGB-recipient mice. (D and E) Body weights (BW) at day 1 and day 21 (D) and OGTT (E) in recipient GF mice transplanted with feces from one RYGB-treated rat and supplemented with heat-killed (*n *= 4) or live (*n *= 4) *L. muris.* *, *P* < 0.05, as determined by two-way ANOVA followed by Bonferroni’s *post hoc* test. (F) Protocol for *L. muris* administration in chow diet-fed and Western-style diet (WSD)-challenged mice. (G and H) Body weights at day 1 and day 21 (G) and OGTT (H) in chow-fed mice administered heat-killed (*n *= 10) or live (*n *= 10) *L. muris.* (I and J) Body weights at day 1 and day 21 (I) and OGTT (J) in WSD-challenged mice administered heat-killed (*n *= 10) or live (*n *= 10) *L. muris*.

In an attempt to characterize a single bacterial species that may contribute to weight loss-independent improvements in glycemic control in RYGB-treated rats, we further focused on the unidentified *Erysipelotrichaceae* species. To reveal its taxonomy, we first filtered all sequences that belong to the family *Erysipelotrichaceae*. We then extracted from this set the sequence whose abundance was strongly reduced in the feces of RYGB-treated rats compared to that of BWM rats. After tabulating the sequences, we performed a BLAST search on the NCBI database. Using this approach, we identified the bacterial species Longibaculum muris as being the most closely related to the unidentified *Erysipelotrichaceae* species. Quantitative PCR further confirmed lower fecal abundance of *L. muris* in RYGB-treated rats than BWM rats (Fig. 2A). Moreover, fecal abundance of *L. muris* correlated positively with the OGTT AUC in RYGB-treated rats (Fig. 2B). Thus, we reasoned that supplementing feces with *L. muris* would reverse the improved oral glucose tolerance in RYGB-recipient mice (Fig. 2C). Unexpectedly, we found that despite no effect on final body weight (Fig. 2D), OGTT showed the opposite outcome; that is, blood glucose levels at 15 min were lower in RYGB-recipient mice supplemented with live *L. muris* than in those receiving heat-killed *L. muris* (Fig. 2E). To determine the impact of *L. muris* alone on host glycemic control under physiological and pathophysiological conditions, we performed experiments on conventionally raised chow-fed and Western style diet-challenged mice, respectively (Fig. 2F). Daily oral gavage of *L. muris* for 3 weeks had minimal impact on body weight (Fig. 2G and I) and on oral glucose tolerance (Fig. 2H and J) under both conditions.

The mechanisms underlying weight loss-independent improvements in glycemic control after metabolic surgery are incompletely understood. In the present study, we confirmed weight loss-independent shifts in the gut microbiota after RYGB, including a reduction in abundances of *Firmicutes* and *Verrucomicrobia* but an expansion in *Proteobacteria* abundances at the phylum level (10, 15, 19). We then showed that fecal *Erysipelotrichaceae* abundance is lower in RYGB-treated rats than in both Sham and BWM rats, which is consistent with the known roles of *Erysipelotrichaceae* species in promoting metabolic dysfunction (18, 20). Moreover, we showed that weight loss-independent improvement in oral glucose tolerance after RYGB is partially transmissible to GF mice via the gut microbiota. Interestingly, only peak glucose levels during the OGTT were lower in RYGB recipient mice, suggesting that the RYGB-associated gut microbiota may contribute specifically to lowering glucose absorption. This is in line with findings from a recent study suggesting that the gut microbiota after metabolic surgery improves oral glucose tolerance by lowering jejunal absorptive capacity rather than by enhancing insulin action (14). Notably, the recipient GF mice in that study also had lower fecal abundance of *Erysipelotrichaceae* family members (14), suggesting a role for this member of the gut microbiota in promoting host glucose absorption.

Accumulating evidence suggests that the gut microbiota regulates host eating behavior and energy balance through its influence on gut-brain communication (21). We found that body weight changes in RYGB recipient mice were not different from those in BWM mice. This contrasts with previous studies showing that transplant of fecal samples from RYGB-treated mice, rats, or humans causes weight loss or protection from weight gain in recipient GF mice (10, 13) or conventionally raised rats with diet-induced obesity (9). A potential explanation for these discrepancies could be the different frequencies of bacterial inoculations and/or the different duration of body weight measurements between studies. Nevertheless, our findings are consistent with the majority of studies showing no effect of gut microbiota transfer from RYGB-treated rats (22) or patients (14, 23, 24) on body weight in GF mice or mice with prior depletion of the gut microbiota from antibiotic treatment (14, 23), rats with prior depletion of the gut microbiota from antibiotic treatment (22), or patients with obesity (24). The findings of the present study thus support the idea that members of the gut microbiota are important contributors to improved glycemic control rather than to weight loss after metabolic surgery, especially in humans (14, 23).

Unexpectedly, we found that providing the *Erysipelotrichaceae* family member *L. muris* as a supplement to RYGB recipient mice further lowered peak glucose levels during an OGTT despite correlating positively with glucose intolerance in RYGB-treated rats. One study showed that *L. muris* is depleted in mice on a low-protein diet compared with mice on a normal-protein diet (25). Therefore, the low abundance of *L. muris* in RYGB-treated rats is probably only a consequence of shifts in protein metabolism (26). These findings not only highlight a dissociation between correlation and causation in gut microbiota studies but also suggest that a multitude of members of the gut microbiota contribute to the improved metabolic phenotype after RYGB rather than any single species. Further defining these bacteria may help in the development of improved noninvasive treatments for metabolic disease that could potentially serve as an adjunct to dieting.

### Animal studies.

**(i) Rat study.** Male Zucker fatty *fa/fa* rats (*n *= 45), aged 6 weeks, were purchased from Charles River, France. They were individually housed in ambient humidity and a temperature of 22°C with a 12-h light/dark cycle with free access to tap water and Purina 5008 Lab diet (Purina Mills, USA; 4.15 kcal/g, 16.7% of kilocalories from fat) unless otherwise stated. Part of the phenotypic data from these animals has previously been reported (15, 16). All experiments were reviewed and approved by the Animal Care Committee of the local government of Unterfranken, Bavaria, Germany (license 55.2–2531.01–72/12). Rats were randomly allocated into three groups: the first group underwent sham surgery (Sham; *n *= 12), the second group underwent RYGB (*n *= 20), and the third group underwent sham surgery and were calorically restricted to match the body weight of the RYGB-treated group (BWM; *n *= 13). Surgeries were performed and perioperative care was implemented as previously described (16). Food intake and body weight were measured for 28 days after surgery. Four RYGB-treated rats were humanely sacrificed due to weight loss within the first 7 postoperative days exceeding tolerable limits. On postoperative day 27, an OGTT was performed at the beginning of the dark cycle as previously described (16). HOMA-IR was calculated by the dividing the product of fasting plasma insulin (in microunits per liter) and blood glucose (in nanomoles per liter) levels by 22.5 (27). The Matsuda-DeFronzo insulin sensitivity index (ISI-M) was calculated based on the results of the OGTT as follows: 10,000/(*G*_0_ × *I*_0_ × *G*_mean_ × *I*_mean_)/2, where *G* and *I* represent blood glucose (in millimoles per deciliter) and plasma insulin (in milliunits per liter) levels, respectively, and “0” and “mean” indicate fasting and mean values, respectively, during the OGTT (28).

On postoperative day 28, rats were terminally anaesthetized with isoflurane/oxygen. Fresh fecal samples were collected (*n *= 12 for Sham rats, *n *= 16 for RYGB-treated rats, and *n *= 5 for BWM rats), snap-frozen in liquid nitrogen, and stored at −80°C.

**(ii) Fecal microbiota transplant.** All mouse experiments were performed at the University of Copenhagen, Denmark, in accordance with the bioethical guidelines, compliant with accepted principles for the care and use of laboratory animals approved by Animal Experiments Inspectorate under the Danish Ministry of Food, Agriculture and Fisheries. Feces from RYGB-treated and BWM rats were suspended in modified brain heart infusion medium supplemented with hemin and l-cysteine as reducing agents. Four-hour-fasting GF Swiss Webster mice taken out of a GF isolator were immediately weighed, subjected to gavage with fecal slurries under a laminar air flow (LAF) bench, and group housed in autoclaved individually ventilated cages in a temperature- and humidity-controlled room at 22°C with 12-h–12-h light/dark cycle. Transplanted mice were given autoclaved normal chow (LabDiet 5010) and water *ad libitum*. After 14 days, an OGTT was performed by oral gavage of glucose (2 g/kg body weight) followed by measurement of blood glucose at the indicated time points. Feces from three donors each of RYGB-treated and BWM rats were transplanted into 3 or 4 GF recipients.

**(iii) Extraction of fecal genomic DNA extraction.** Total genomic DNA was extracted from 60 to 80 mg of feces of rats or mice using repeated bead beating (29). Briefly, samples were placed in lysing matrix E tubes (MP Biomedicals) and extracted twice in lysis buffer (4% [wt/vol] sodium dodecyl sulfate, 500 mmol/L NaCl, 50 mmol/L EDTA, 50 mmol/L Tris hydrochloride [pH 8]) with bead beating at 5.0 m/s for 60 s in a FastPrep 24 instrument (MP Biomedicals). After each bead-beating cycle, samples were incubated at 95°C for 15 min and then centrifuged at full speed for 5 min at 4°C. Supernatants from the two extractions were pooled, and the DNA was recovered by isopropanol purification and then purified using the QIAamp DNA minikit (Qiagen).

### 16S rRNA gene amplification, sequencing, and data analysis.

The V4 variable region of the 16S rRNA genes from each sample was amplified with 515F and 806R primers, designed for dual indexing (30) in duplicate reactions. PCR amplification was performed in a 25-mL volume containing AccuPrime Pfx SuperMix (Invitrogen), a 200 nM concentration of each primer, and 20 ng of genomic DNA. PCR was carried out by initial denaturation for 3 min at 95°C, followed by 25 cycles (denaturation for 45 s at 95°C, annealing for 60 s at 52°C, and elongation for 90 s at 72°C) and a final elongation step for 10 min at 72°C. Duplicates were combined, purified with the NucleoSpin gel and PCR clean-up kit (Macherey-Nagel, Germany), and quantified using the Qubit double-strand DNA (dsDNA) HS assay kit (Invitrogen). The amplified V4 region of the 16S rRNA gene was sequenced with 250-bp paired-end reads on an Illumina MiniSeq instrument (RTA v. 2.11.4.0; MCS 2.0.0.16) with the MiniSeq Mid output kit in two batches.

Sequencing data were analyzed using Quantitative Insights into Microbial Ecology 2 (QIIME 2) (31). Paired-end reads were merged and quality and chimera filtered using DADA2 (32). Taxonomy was assigned using the Ribosomal Database Project Classifier (33) against the 16S rRNA gene reference GreenGenes database (v.13.8) (34). We obtained a total of 3,756,593 reads, and 1,170 operational taxonomic units (OTUs) were included in the analyses. Genus- and species-level analysis of the abundance on OTUs collapsed to the same genus (L6 level) and species (L7 level) was carried out. To correct for differences in sequencing depth, samples were subsampled to the same number of reads (37,000 reads). In analysis of relative abundance on genus (L6-level) and species (L7-level) counts, the number of counts was scaled to the total sum of counts. The values given as relative abundances sum to 1. Analysis of compositions of microbiomes with bias correction (ANCOM-BC) (35) was applied to identify the differential absolute abundance of microbes in the different groups.

To delineate the species identity of the unidentified *Erysipelotrichaceae* species, we first filtered the sequences belonging to the family *Erysipelotrichaceae* from the 16S rRNA sequencing data in a table using the filter-seqs in the q2-taxa plugin. Then, we extracted only the sequences that had low relative abundance in the RYGB rats compared to the BWM rats. After tabulation of the sequences (qiime feature-table tabulate-seqs), the sequences were directly searched in the NCBI nucleotide BLAST browser. The unidentified *Erysipelotrichaceae* species was identified with 97.2% identity as Longibaculum muris. The full 16S rRNA sequence of the only available cultivable *L. muris* DSM 29487 strain was used to design primers for quantitative PCR (qPCR) that target the V4 region using the NCBI Primer-BLAST tool.

### Bacterial culture and *in vivo* administration.

Longibaculum muris DSM 29487 used in the study was of mouse origin and was obtained from DSMZ (Braunschweig, Germany). The strain was grown in yeast casitone fatty acids (YCFA) broth (36) at 37°C anaerobically in 15-mL Hungate tubes. Fresh *L. muris* culture was subcultured anaerobically a day before administration to mice via gavage. To obtain heat-killed bacteria, *L. muris* was cultured and autoclaved at 121°C for 15 min. The tubes containing heat-killed bacteria were tested for no growth and stored at 4°C until further use. Two independent *in vivo* administration experiments were performed.

First, fecal slurry from one donor of RYGB-treated rats was orally administered via gavage to GF mice as described above. After 1 week of transplantation, RYGB-transplanted mice were randomly assigned to receive 200 μL of fresh or heat-killed *L. muris* culture for 3 weeks. An OGTT was performed and blood glucose measured at the indicated time points.

For the second experiment, C57BL/6 mice (*n *= 10) were procured from Janvier, Denmark, and group housed in individually ventilated cages in a temperature- and humidity-controlled room at 22°C with a 12-h–12-h light/dark cycle. Mice were acclimatized for 1 week prior to the start of the experiment and then randomly allocated to receive 200 μL of either fresh or heat-killed bacteria twice a week by oral gavage for 3 weeks. All mice were given normal chow (LabDiet 5010) and water *ad libitum*. An OGTT was performed by oral gavage of glucose (2 g/kg body weight), and subsequently, blood glucose was measured at the indicated time points. After OGTT was performed for mice on normal chow, the diet of both groups of mice was shifted to a Western-style diet (Research Diets; D12451), and mice continued to receive gavage with 200 μL of either fresh or heat-killed bacteria twice a week for 3 weeks. An OGTT was again performed and blood glucose measured at the indicated time points.

### 16S rRNA quantitative PCR.

16S rRNA quantitative PCR was performed with a CFX96 real-time system (Bio-Rad). All reactions were performed in duplicate in one run and in duplicate PCR runs. Samples were analyzed in a 10-μL reaction mix consisting of 6.5 μL 1× SYBR green master mix buffer (Thermo Scientific, Waltham, MA, USA), a 0.2 μM concentration of each primer, and 1 μL of template DNA, water, or genomic DNA extracted from feces. Standard curves of the full 16S rRNA PCR product of *L. muris* were created using serial 10-fold dilution of the purified PCR product. The following primers were used to quantify *L. muris* 16S rRNA copies: Lm forward (internal number, JVO 21060), CAGACGGGGACAACGATTGGA; Lm reverse (internal number, JVO 21061), ACGCATCGTCGCCTTGGTA.

### Statistical analysis.

The Mann-Whitney U test, an unpaired, two-tailed *t* test, one-way analysis of variance (ANOVA) followed by Holm-Šidák’s *post hoc* test, and two-way ANOVA followed by Bonferroni’s *post hoc* test were performed using GraphPad Prism software (version 9.3) where indicated. Permutational multivariate ANOVA (PERMANOVA) followed by a pairwise *post hoc* test, Kruskal-Wallis test corrected for false discovery rate (FDR) by the Benjamini-Hochberg method, and Spearman’s correlation analysis of metabolic features (OGTT AUC, HOMA-IR, ISI-M, body weight (BW) and food intake (FI)) were performed using R statistical analysis software version 4.1.2 (http://www.R-project.org/) where indicated. Adjusted and raw *P* values of <0.05 were considered statistically significant.

### Data availability.

All 16S rRNA gene sequencing data included in this study have been deposited in the European Nucleotide Archive (ENA). The data for the Zucker fatty rats are available under accession no. PRJEB58153 and the data for the mice under accession no. PRJEB58198.

## References

[1] Afshin A, Forouzanfar MH, Reitsma MB, Sur P, Estep K, Lee A, Marczak L, Mokdad AH, Moradi-Lakeh M, Naghavi M, Salama JS, Vos T, Abate KH, Abbafati C, Ahmed MB, Al-Aly Z, Alkerwi A, Al-Raddadi R, Amare AT, Amberbir A, Amegah AK, Amini E, Amrock SM, Anjana RM, Arnlov J, Asayesh H, Banerjee A, Barac A, Baye E, Bennett DA, Beyene AS, Biadgilign S, Biryukov S, Bjertness E, Boneya DJ, Campos-Nonato I, Carrero JJ, Cecilio P, Cercy K, Ciobanu LG, Cornaby L, Damtew SA, Dandona L, Dandona R, Dharmaratne SD, Duncan BB, Eshrati B, Esteghamati A, Feigin VL, Fernandes JC. GBD 2015 Obesity Collaborators, et al. 2017. Health effects of overweight and obesity in 195 countries over 25 years. N Engl J Med 377:13–27. doi:10.1056/NEJMoa1614362. 28604169 PMC5477817

[2] Bluher M. 2019. Obesity: global epidemiology and pathogenesis. Nat Rev Endocrinol 15:288–298. doi:10.1038/s41574-019-0176-8. 30814686

[3] Turnbaugh PJ, Ley RE, Mahowald MA, Magrini V, Mardis ER, Gordon JI. 2006. An obesity-associated gut microbiome with increased capacity for energy harvest. Nature 444:1027–1031. doi:10.1038/nature05414. 17183312

[4] Muller TD, Bluher M, Tschop MH, DiMarchi RD. 2022. Anti-obesity drug discovery: advances and challenges. Nat Rev Drug Discov 21:201–223. doi:10.1038/s41573-021-00337-8. 34815532 PMC8609996

[5] Evers SS, Sandoval DA, Seeley RJ. 2017. The physiology and molecular underpinnings of the effects of bariatric surgery on obesity and diabetes. Annu Rev Physiol 79:313–334. doi:10.1146/annurev-physiol-022516-034423. 27912678

[6] Albaugh VL, He Y, Munzberg H, Morrison CD, Yu S, Berthoud HR. 2022. Regulation of body weight: lessons learned from bariatric surgery. Mol Metab 68:101517. doi:10.1016/j.molmet.2022.101517. 35644477 PMC9938317

[7] Dang JT, Mocanu V, Park H, Laffin M, Hotte N, Karmali S, Birch DW, Madsen KL. 2022. Roux-en-Y gastric bypass and sleeve gastrectomy induce substantial and persistent changes in microbial communities and metabolic pathways. Gut Microbes 14:2050636. doi:10.1080/19490976.2022.2050636. 35316158 PMC8942407

[8] Jahansouz C, Staley C, Kizy S, Xu H, Hertzel AV, Coryell J, Singroy S, Hamilton M, DuRand M, Bernlohr DA, Sadowsky MJ, Khoruts A, Ikramuddin S. 2019. Antibiotic-induced disruption of intestinal microbiota contributes to failure of vertical sleeve gastrectomy. Ann Surg 269:1092–1100. doi:10.1097/SLA.0000000000002729. 31082907

[9] Munzker J, Haase N, Till A, Sucher R, Haange SB, Nemetschke L, Gnad T, Jager E, Chen J, Riede SJ, Chakaroun R, Massier L, Kovacs P, Ost M, Rolle-Kampczyk U, Jehmlich N, Weiner J, Heiker JT, Kloting N, Seeger G, Morawski M, Keitel V, Pfeifer A, von Bergen M, Heeren J, Krugel U, Fenske WK. 2022. Functional changes of the gastric bypass microbiota reactivate thermogenic adipose tissue and systemic glucose control via intestinal FXR-TGR5 crosstalk in diet-induced obesity. Microbiome 10:96. doi:10.1186/s40168-022-01264-5. 35739571 PMC9229785

[10] Liou AP, Paziuk M, Luevano JM, Jr, Machineni S, Turnbaugh PJ, Kaplan LM. 2013. Conserved shifts in the gut microbiota due to gastric bypass reduce host weight and adiposity. Sci Transl Med 5:178ra41. doi:10.1126/scitranslmed.3005687. PMC3652229 23536013

[11] Hankir MK, Langseder T, Bankoglu EE, Ghoreishi Y, Dischinger U, Kurlbaum M, Kroiss M, Otto C, Le Roux CW, Arora T, Seyfried F, Schlegel N. 2020. Simulating the post-gastric bypass intestinal microenvironment uncovers a barrier-stabilizing role for FXR. iScience 23:101777. doi:10.1016/j.isci.2020.101777. 33294786 PMC7689555

[12] Arora T, Seyfried F, Docherty NG, Tremaroli V, Le Roux CW, Perkins R, Backhed F. 2017. Diabetes-associated microbiota in fa/fa rats is modified by Roux-en-Y gastric bypass. ISME J 11:2035–2046. doi:10.1038/ismej.2017.70. 28524868 PMC5563957

[13] Tremaroli V, Karlsson F, Werling M, Stahlman M, Kovatcheva-Datchary P, Olbers T, Fandriks L, Le Roux CW, Nielsen J, Backhed F. 2015. Roux-en-Y gastric bypass and vertical banded gastroplasty induce long-term changes on the human gut microbiome contributing to fat mass regulation. Cell Metab 22:228–238. doi:10.1016/j.cmet.2015.07.009. 26244932 PMC4537510

[14] Anhe FF, Zlitni S, Zhang SY, Choi BS, Chen CY, Foley KP, Barra NG, Surette MG, Biertho L, Richard D, Tchernof A, Lam TKT, Marette A, Schertzer J. 2022. Human gut microbiota after bariatric surgery alters intestinal morphology and glucose absorption in mice independently of obesity. Gut 72:460–471. doi:10.1136/gutjnl-2022-328185. 36008102 PMC9933168

[15] Seyfried F, Phetcharaburanin J, Glymenaki M, Nordbeck A, Hankir M, Nicholson JK, Holmes E, Marchesi JR, Li JV. 2021. Roux-en-Y gastric bypass surgery in Zucker rats induces bacterial and systemic metabolic changes independent of caloric restriction-induced weight loss. Gut Microbes 13:1875108. doi:10.1080/19490976.2021.1875108. 33535876 PMC7872092

[16] Seyfried F, Miras AD, Rotzinger L, Nordbeck A, Corteville C, Li JV, Schlegel N, Hankir M, Fenske W, Otto C, Jurowich C. 2016. Gastric bypass-related effects on glucose control, beta cell function and morphology in the obese Zucker rat. Obes Surg 26:1228–1236. doi:10.1007/s11695-015-1882-5. 26377340

[17] Hankir MK, Seyfried F. 2020. Do bariatric surgeries enhance brown/beige adipose tissue thermogenesis? Front Endocrinol 11:275. doi:10.3389/fendo.2020.00275. PMC7203442 32425889

[18] Kaakoush NO. 2015. Insights into the role of Erysipelotrichaceae in the human host. Front Cell Infect Microbiol 5:84. doi:10.3389/fcimb.2015.00084. 26636046 PMC4653637

[19] Haange SB, Jehmlich N, Krugel U, Hintschich C, Wehrmann D, Hankir M, Seyfried F, Froment J, Hubschmann T, Muller S, Wissenbach DK, Kang K, Buettner C, Panagiotou G, Noll M, Rolle-Kampczyk U, Fenske W, von Bergen M. 2020. Gastric bypass surgery in a rat model alters the community structure and functional composition of the intestinal microbiota independently of weight loss. Microbiome 8:13. doi:10.1186/s40168-020-0788-1. 32033593 PMC7007695

[20] Truax AD, Chen L, Tam JW, Cheng N, Guo H, Koblansky AA, Chou WC, Wilson JE, Brickey WJ, Petrucelli A, Liu R, Cooper DE, Koenigsknecht MJ, Young VB, Netea MG, Stienstra R, Sartor RB, Montgomery SA, Coleman RA, Ting JP. 2018. The inhibitory innate immune sensor NLRP12 maintains a threshold against obesity by regulating gut microbiota homeostasis. Cell Host Microbe 24:364–378.E6. doi:10.1016/j.chom.2018.08.009. 30212649 PMC6161752

[21] de Wouters d’Oplinter A, Huwart SJP, Cani PD, Everard A. 2022. Gut microbes and food reward: from the gut to the brain. Front Neurosci 16:947240. doi:10.3389/fnins.2022.947240. 35958993 PMC9358980

[22] Liu Z, Coales I, Penney N, McDonald JAK, Phetcharaburanin J, Seyfried F, Li JV. 2020. A subset of Roux-en-Y gastric bypass bacterial consortium colonizes the gut of nonsurgical rats without inducing host-microbe metabolic changes. mSystems 5:e01047-20. doi:10.1128/mSystems.01047-20. 33293406 PMC8579838

[23] Debedat J, Le Roy T, Voland L, Belda E, Alili R, Adriouch S, Bel Lassen P, Kasahara K, Hutchison E, Genser L, Torres L, Gamblin C, Rouault C, Zucker JD, Kapel N, Poitou C, Marcelin G, Rey FE, Aron-Wisnewsky J, Clement K. 2022. The human gut microbiota contributes to type-2 diabetes non-resolution 5-years after Roux-en-Y gastric bypass. Gut Microbes 14:2050635. doi:10.1080/19490976.2022.2050635. 35435140 PMC9037437

[24] de Groot P, Scheithauer T, Bakker GJ, Prodan A, Levin E, Khan MT, Herrema H, Ackermans M, Serlie MJM, de Brauw M, Levels JHM, Sales A, Gerdes VE, Stahlman M, Schimmel AWM, Dallinga-Thie G, Bergman JJ, Holleman F, Hoekstra JBL, Groen A, Backhed F, Nieuwdorp M. 2020. Donor metabolic characteristics drive effects of faecal microbiota transplantation on recipient insulin sensitivity, energy expenditure and intestinal transit time. Gut 69:502–512. doi:10.1136/gutjnl-2019-318320. 31147381 PMC7034343

[25] Masuoka H, Suda W, Tomitsuka E, Shindo C, Takayasu L, Horwood P, Greenhill AR, Hattori M, Umezaki M, Hirayama K. 2020. The influences of low protein diet on the intestinal microbiota of mice. Sci Rep 10:17077. doi:10.1038/s41598-020-74122-9. 33051527 PMC7555506

[26] Bojsen-Moller KN, Jacobsen SH, Dirksen C, Jorgensen NB, Reitelseder S, Jensen JE, Kristiansen VB, Holst JJ, van Hall G, Madsbad S. 2015. Accelerated protein digestion and amino acid absorption after Roux-en-Y gastric bypass. Am J Clin Nutr 102:600–607. doi:10.3945/ajcn.115.109298. 26245808

[27] Matthews DR, Hosker JP, Rudenski AS, Naylor BA, Treacher DF, Turner RC. 1985. Homeostasis model assessment: insulin resistance and beta-cell function from fasting plasma glucose and insulin concentrations in man. Diabetologia 28:412–419. doi:10.1007/BF00280883. 3899825

[28] Matsuda M, DeFronzo RA. 1999. Insulin sensitivity indices obtained from oral glucose tolerance testing: comparison with the euglycemic insulin clamp. Diabetes Care 22:1462–1470. doi:10.2337/diacare.22.9.1462. 10480510

[29] Salonen A, Nikkila J, Jalanka-Tuovinen J, Immonen O, Rajilic-Stojanovic M, Kekkonen RA, Palva A, de Vos WM. 2010. Comparative analysis of fecal DNA extraction methods with phylogenetic microarray: effective recovery of bacterial and archaeal DNA using mechanical cell lysis. J Microbiol Methods 81:127–134. doi:10.1016/j.mimet.2010.02.007. 20171997

[30] Kozich JJ, Westcott SL, Baxter NT, Highlander SK, Schloss PD. 2013. Development of a dual-index sequencing strategy and curation pipeline for analyzing amplicon sequence data on the MiSeq Illumina sequencing platform. Appl Environ Microbiol 79:5112–5120. doi:10.1128/AEM.01043-13. 23793624 PMC3753973

[31] Bolyen E, Rideout JR, Dillon MR, Bokulich NA, Abnet CC, Al-Ghalith GA, Alexander H, Alm EJ, Arumugam M, Asnicar F, Bai Y, Bisanz JE, Bittinger K, Brejnrod A, Brislawn CJ, Brown CT, Callahan BJ, Caraballo-Rodríguez AM, Chase J, Cope EK, Da Silva R, Diener C, Dorrestein PC, Douglas GM, Durall DM, Duvallet C, Edwardson CF, Ernst M, Estaki M, Fouquier J, Gauglitz JM, Gibbons SM, Gibson DL, Gonzalez A, Gorlick K, Guo J, Hillmann B, Holmes S, Holste H, Huttenhower C, Huttley GA, Janssen S, Jarmusch AK, Jiang L, Kaehler BD, Kang KB, Keefe CR, Keim P, Kelley ST, Knights D, et al. 2019. Reproducible, interactive, scalable and extensible microbiome data science using QIIME 2. Nat Biotechnol 37:852–857. doi:10.1038/s41587-019-0209-9. 31341288 PMC7015180

[32] Callahan BJ, McMurdie PJ, Rosen MJ, Han AW, Johnson AJ, Holmes SP. 2016. DADA2: high-resolution sample inference from Illumina amplicon data. Nat Methods 13:581–583. doi:10.1038/nmeth.3869. 27214047 PMC4927377

[33] Wang Q, Garrity GM, Tiedje JM, Cole JR. 2007. Naive Bayesian classifier for rapid assignment of rRNA sequences into the new bacterial taxonomy. Appl Environ Microbiol 73:5261–5267. doi:10.1128/AEM.00062-07. 17586664 PMC1950982

[34] DeSantis TZ, Hugenholtz P, Larsen N, Rojas M, Brodie EL, Keller K, Huber T, Dalevi D, Hu P, Andersen GL. 2006. Greengenes, a chimera-checked 16S rRNA gene database and workbench compatible with ARB. Appl Environ Microbiol 72:5069–5072. doi:10.1128/AEM.03006-05. 16820507 PMC1489311

[35] Lin H, Peddada SD. 2020. Analysis of compositions of microbiomes with bias correction. Nat Commun 11:3514. doi:10.1038/s41467-020-17041-7. 32665548 PMC7360769

[36] Ze X, Duncan SH, Louis P, Flint HJ. 2012. Ruminococcus bromii is a keystone species for the degradation of resistant starch in the human colon. ISME J 6:1535–1543. doi:10.1038/ismej.2012.4. 22343308 PMC3400402

