# Supplementary material for: Gut Microbiota Contribution to Weight-Independent Glycemic Improvements after Gastric Bypass Surgery
Source: Microbiol Spectr. 2023 Apr 6;11(3):e05109-22. doi: 10.1128/spectrum.05109-22 (PMC10269853; doi:10.1128/spectrum.05109-22)
Supplement: Supplemental file 1 — Supplemental material. Download spectrum.05109-22-s0001.pdf, PDF file, 0.3 MB [file spectrum.05109-22-s0001.pdf]

	Sham (*n* = 12)	RYGB (*n* = 16)	BWM (*n* = 13)
OGTT AUC	1,343 ± 83.8	871 ± 22.6****	976 ± 27.1****,$^{\#\#}$
HOMA-IR	53.4 ± 4.8	16.4 ± 3.1****	48.6 ± 6.3^{+++}
ISI-M	0.03 ± 0.003	0.08 ± 0.015**	0.04 ± 0.004^{++}
Body weight (g) at postoperative day 28	559 ± 8.6	424 ± 11.6****	431 ± 1.5****
Food intake (g) at postoperative day 27	32 ± 0.7	22 ± 1.3****	18 ± 0.3****,$^{++}$

Supplementary Table 1. Metabolic phenotype of Zucker fatty rats used in this study. ****$P < 0.0001$ and **$P < 0.01$ for Sham vs RYGB and for Sham vs BWM as determined by one-way ANOVA followed by Holm-Sidak's posthoc test. $^{+++}P < 0.001$ and $^{++}P < 0.01$ for RYGB vs BWM as determined by one-way ANOVA followed by Holm-Sidak's posthoc test. $^{\#\#}P < 0.01$ for RYGB vs BWM as determined by unpaired, two-tailed *t*-test.

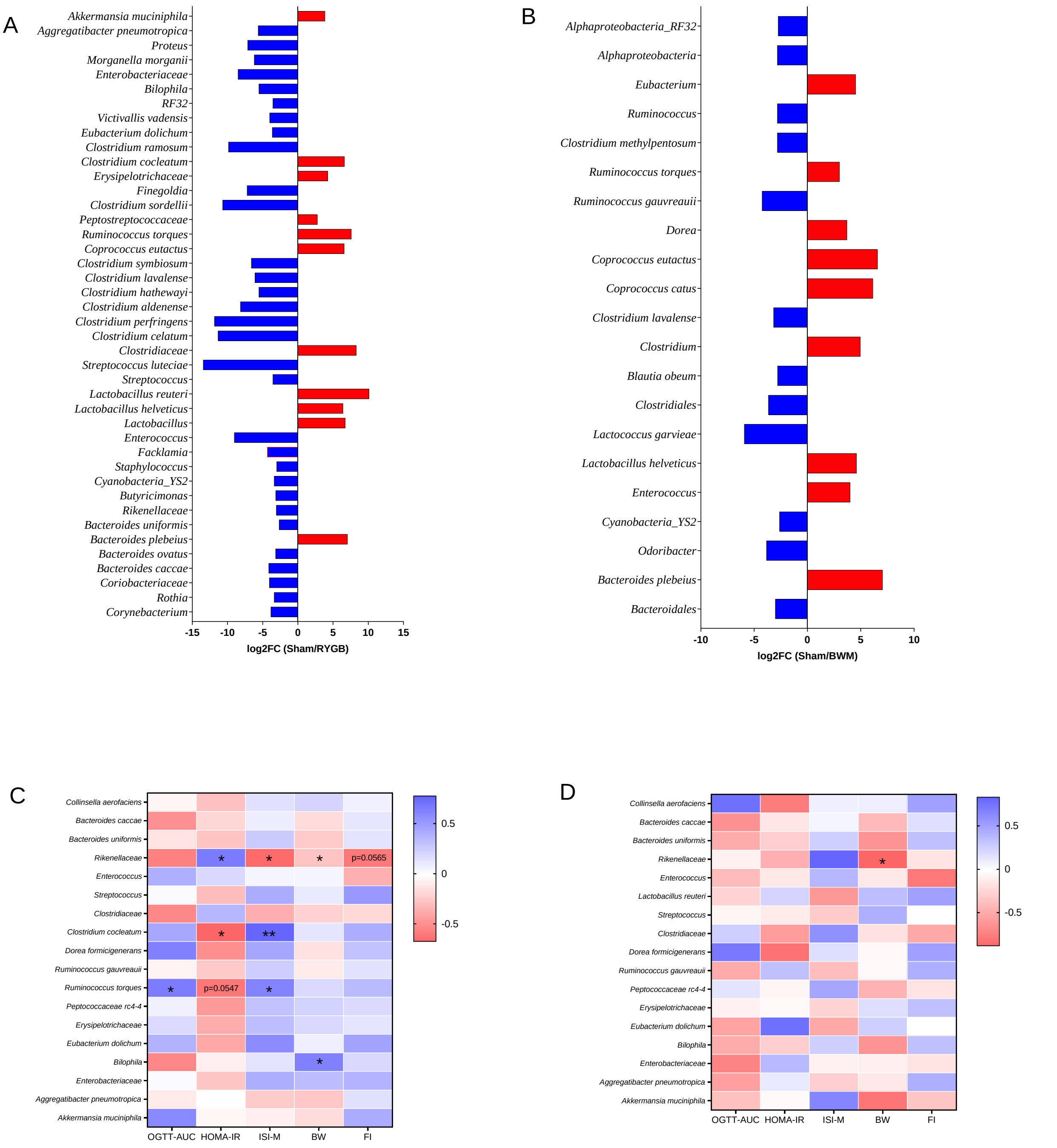

Supplementary Figure 1 (related to Figure 1). Differential species expressed as log2 fold-change in A) Sham versus RYGB-treated rats and B) Sham versus BWM rats. Correlation matrix of species with metabolic parameters in C) Sham and D) BWM rats. ***Q* < 0.01 and **Q* < 0.05 as determined by Mann-Whitney U test after adjusting for multiple comparisons.
